# Temporal and Spatial Dynamics of Motor Dysfunction in Preclinical Parkinson’s Disease and Aging

**DOI:** 10.20900/jpbs.20250009

**Published:** 2025-08-06

**Authors:** Navya Nair, Grace Hey, Alexander Becsey, Tara Kari, Xavier Becsey, Julia Root, Vinata Vedam-Mai

**Affiliations:** 1 Lillian S. Wells Department of Neurosurgery, College of Medicine, University of Florida, Gainesville, FL 32611, USA; 2 Norman Fixel Institute for Neurological Diseases, College of Medicine, University of Florida, Gainesville, FL 32608, USA

**Keywords:** Parkinson’s disease, murine model, gait, M83^+/−^

## Abstract

Gait assessments have been performed in several murine models of Parkinson’s Disease (PD), but the M83^+/−^ mouse model of PD has been relatively understudied in this context. Metrics of gait swing, stride length and frequency, and ataxia were collected in M83^+/−^ mice with peripheral injections of α-syn preformed fibrils (PFF) and in aged M83^+/−^ mice without fibrils using the DigiGait^™^ system. PFF-mice showed significantly decreased swing in all limbs (0.11 ± 0.02 vs. 0.13 ± 0.03, *p* = 0.007) compared to age-matched controls. Stride frequency was significantly increased in all limbs (3.9 ± 0.4 vs. 3.0 ± 0.5, *p* = 0.010) of PFF-treated mice. Swing was significantly greater in the hindlimbs of young M83^+/−^+PFF mice compared to aged M83^+/−^ mice (0.11(0.5) vs. 0.08 (0.3), *p* = 0.015). Ataxia was significantly higher in young M83^+/−^+PFF mice compared to control for forelimbs (1.1 ± 1.0 vs. 0.6 ± 0.5, *p* = 0.027), hindlimbs (0.9 ± 1.0 vs. 0.3 ± 0.2, *p* = 0.016), and all limbs (1.0 ± 1.0 vs. 0.3 ± 0.5, *p* = 0.015). M83^+/−^ mice demonstrate significant gait abnormalities consistent with features of PD. This study supports the utility of the M83^+/−^ murine model for preclinical gait analyses in PD.

## INTRODUCTION

Walking is a complex motor behavior that requires precise control of initiation and termination of locomotion, dynamic adjustments in speed, stride length, direction, and posture in response to both internal and external cues [1]. Degeneration of the central or peripheral nervous system(s) (CNS or PNS) can significantly impair one’s ability to locomote successfully. PD is a chronic, neurodegenerative disorder characterized by the loss of dopaminergic neurons in the substantia nigra. The histopathological hallmark of PD is the presence of misfolded alpha-synuclein (α-syn) into Lewy bodies, which results in neuronal demise that manifests as classic motor symptoms including bradykinesia, rigidity, resting tremor, and postural instability [2].

PD patients tend to exhibit characteristic gait abnormalities which may be categorized as either continuous (persistent) or episodic (freezing of gait, festination). Continuous gait disturbances are usually studied more due to the dynamic nature of the issue. Some of these disturbances include higher step-to-step variability and stride length variability [3,4]. These changes in gait can be observed at the time of diagnosis and monitored over time through use of kinematic, spatial, or temporal measures such as video-analysis, footfall studies, or wearable devices. Although advancements in imaging technology allow for visualization of circuitries involved in control of gait, treatment strategies that target specific gait difficulties are restricted by a limited understanding of their functional-anatomical basis [5-10]. Furthermore, human gait dynamics are exceedingly complex, involving several neuronal circuits as well as high-order spatial and temporal indices including angle of foot placement, stance width stride frequency, stride duration, and swing duration [3,4,11,12].

Murine models are often used to assess the neurobiology and pathophysiology of gait dynamics in PD. Of note, the M83^+/−^ transgenic mouse model which overexpresses human α-syn, with a point mutation in the *SNCA* gene at the 53^rd^ position resulting in the replacement of the Alanine with Threonine (A53T) [13]. As a result, there is an alteration in the conformation of the α-syn protein, which causes it to become prone to misfolding and aggregation. It is these abnormal aggregates that are thought to be central to the development of Parkinson’s disease and are found in Lewy bodies, which are pathological hallmarks of PD [14]. Although behavioral assessments have been performed in several murine models of PD, the M83^+/−^ mouse model has been relatively understudied in the context of gait [15-18]. The purpose of this study is to report on the temporal and spatial indices of gait dynamics in M83^+/−^ mice which can help inform us on important circuit contributors to overall locomotor activity, which will aid in clinical and translational opportunities to alleviate gait issues in PD.

## METHODS

### Animals and Groups

All animals (N = 48) were maintained on a 12-hour light 12-hour dark schedule with ad libitum access to food and water. Handling, care of mice, and all animal experiments were approved by the University of Florida Institutional Animal Care and Use Committee (UF IACUC).

### α-syn Injections

At around two months of age, human Ala53Thr hemizygous (M83^+/−^) α-syn transgenic mice obtained as a gift from Dr. Benoit Giasson, maintained at the UF animal breeding core (PMID 12062037) received a one-time bilateral intramuscular (biceps femoris) injection of 10 μg of full-length (wild-type) mouse α-syn fibrils or 5 μL of sterile PBS [19]. M83^+/−^ Tg mice were maintained on a C57Bl/C3H background strain. Both groups contained equal numbers of males and females.

**Figure 1.**
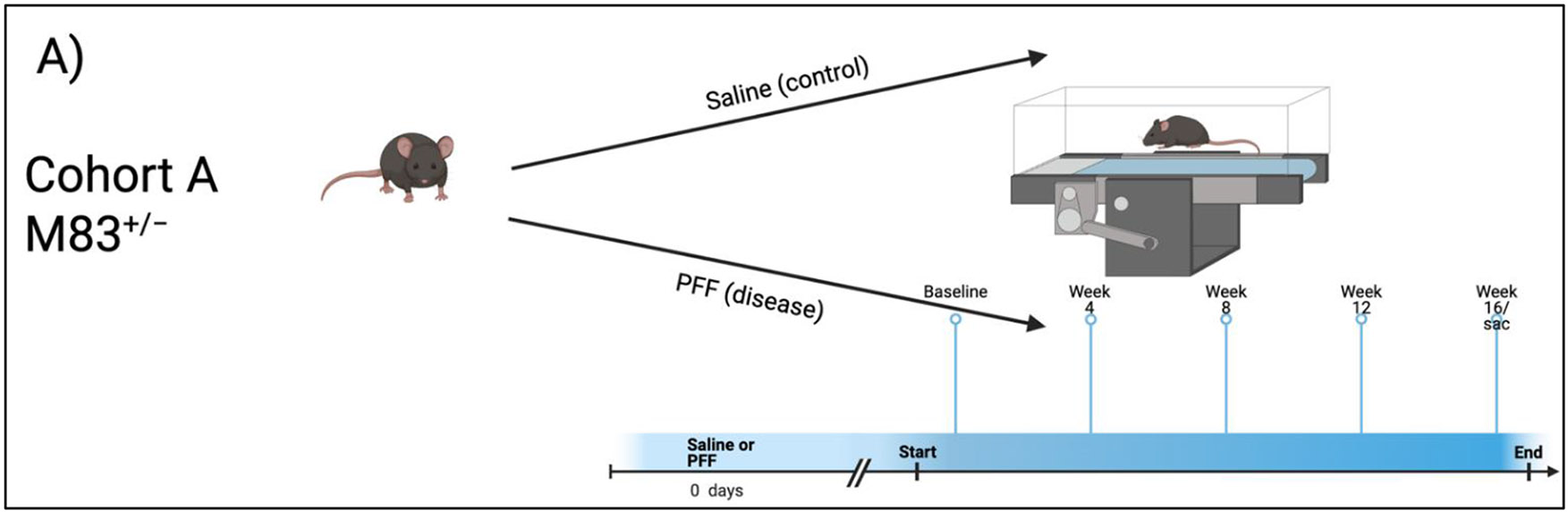

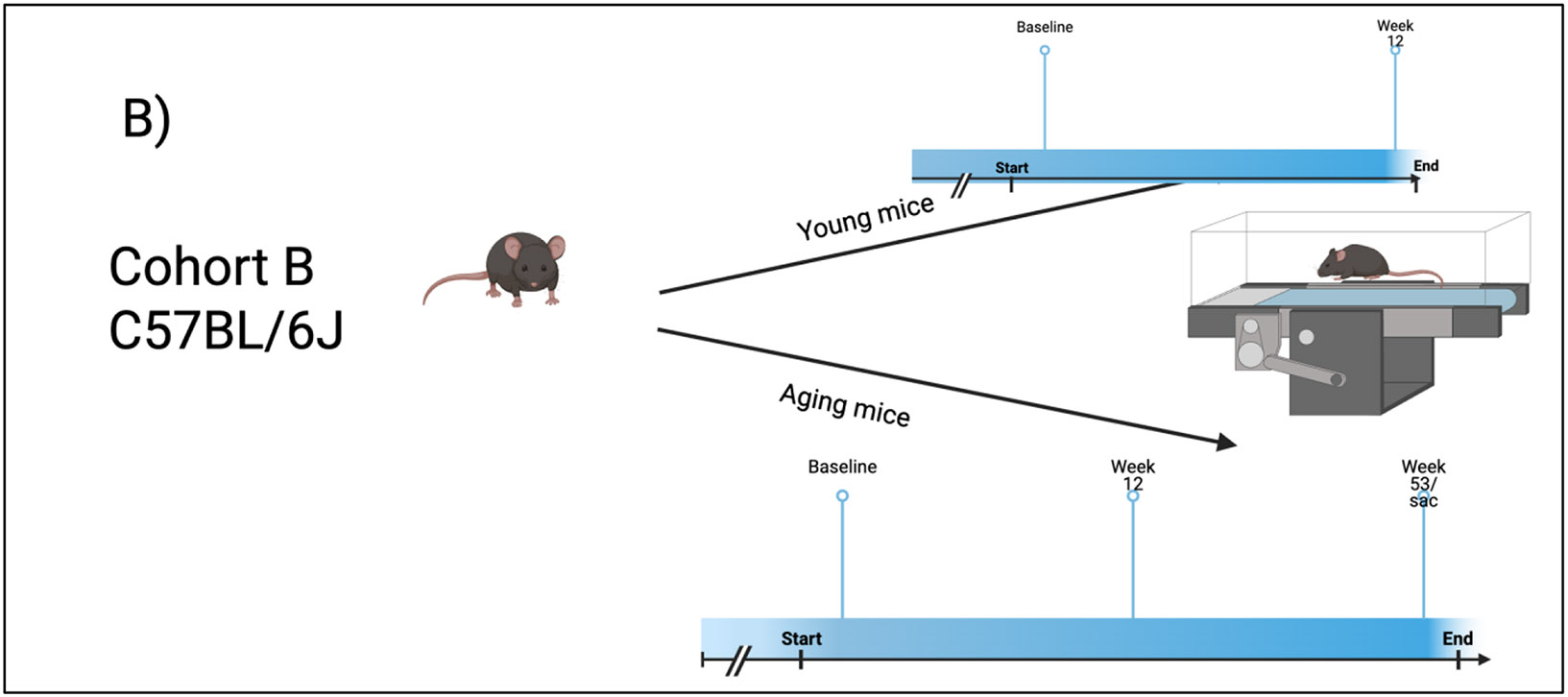
Experimental design. (**A**) Outlines the protocol for cohort A. (**B**) Outlines the protocol for cohort B.

Peripheral, intramuscular injections of PFFs were administered as described previously to ensure rapid and synchronous induction of widespread α-syn pathology. Part I of this study was designed to evaluate the change in gait behaviors across a 16-week period in PFF-injected M83^+/−^ mice (Cohort A, N = 19, Figure 1A). 8-week-old M83^+/−^ mice (N = 12; 7 female, 5 male) were anesthetized and injected with 10 μg of hfib21-140 α-syn PFFs per hind leg (α-syn). 8-week-old M83^+/−^ mice (N = 7; 4 female, 3 male) were injected with equal amounts of saline (control). Various gait metrics were recorded at 5 different time points: baseline/start of the study, 4-, 8-, 12- and 16-weeks post PFF injections.

Part II of this study was designed to assess the differences in gait dynamics due to aging in between M83^+/−^ mice not injected with PFF (N = 7; 4 male and 3 female) at a “young” age (12-13-week-old) and “aged” M83^+/−^ mice without PFF (N = 6; 3 male and 3 female) at a later timepoint (53-54-week-old) (Cohort B, N = 29, Figure 1B). Age-matched 12-13-week-old (N = 8; all male) and 53-54-week-old (N = 8; 5 female and 3 male) C57BL/6J mice served as the control for the two groups respectively in this analysis.

### Gait Dynamics

Gait evaluations were performed on all mice in both parts I and II of this study. Gait dynamics were recorded using the DigiGait^™^ (Mouse Specifics, Inc., Framingham, MA, USA) treadmill system with ventral plane videography. For both groups, mice were acclimated by running up to 20.0 cm/s for 30 s. All mice were then run at 20.0 cm/s at a uniform speed between the boundaries of the compartment within the machine. Any measurement not recorded at 20 cm/s were excluded from the study. Any footage where the mouse stopped, jumped, or touched the front or back of the compartment was removed. For the M83^+/−^+PFF mice, gait metrics were collected every four weeks following injection of PFF/phosphate-buffered saline. For mice in Cohort A, gait metrics were collected at baseline, four weeks, eight weeks, twelve weeks, and sixteen weeks of age after PFF injections at 0° incline. The recording duration was between 3.5 and 4 s. For aged mice, gait metrics were collected at 12 and 53 weeks of age at 0° incline for all timepoints.

**Figure 2.**
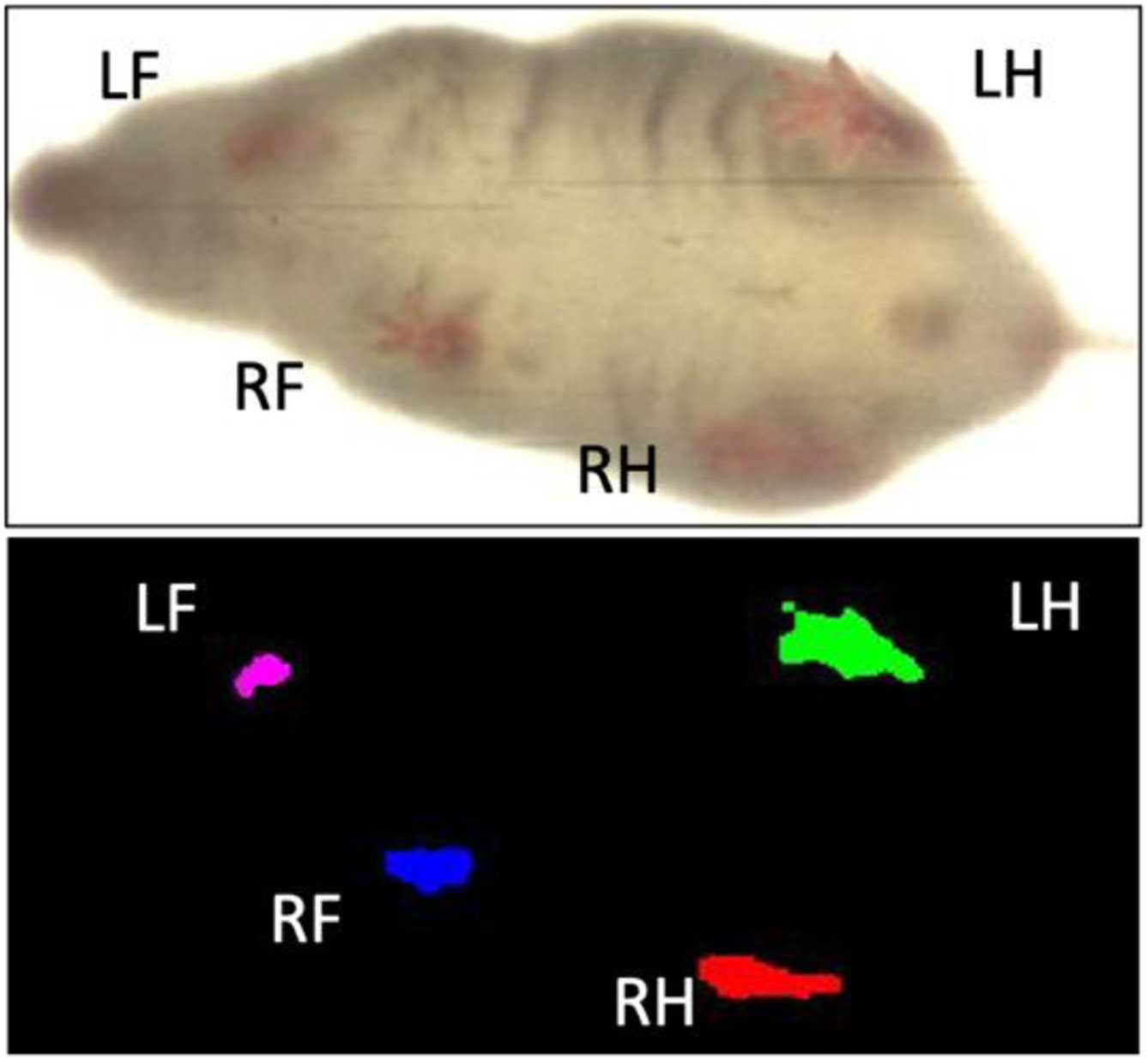
Ventral view of a walking mouse analyzed with DigiGait^™^ recording software. This image represents the transformed digital paw prints of a mouse walking on the DigiGait^™^ treadmill at a speed of 20 cm/s. (Mouse Specifics, Inc., 2019).

Data was then analyzed using DigiGait software V15. The nose and tail of the mouse was manually cropped, and hair color was filtered to allow for easy paw detection. Each paw was tracked frame by frame to assess how often and how much contact the paw had with the treadmill. Ventral plane imaging technology was used to automatically calculate paw contact surface area and paw direction of contact. Dynamic gait signals are then generated that display paw area over time, as seen in Figure 2. Where applicable, compression artifacts and errors were removed.

For this study, gait dynamics are classified into three domains—forelimbs, hindlimbs, and all limbs. Forelimb data was obtained by finding the combined average of right front paw and left front paw data. Hindlimb data was obtained by calculating the combined average of right hind paw and left hind paw data. The term “all limbs” refers to the combined average of data from all four paws.

### Statistics

Two-way analysis of variance (ANOVA) with Sidak’s multiple comparisons was used to compare differences in swing, stride frequency, stride length, stride length variability, and ataxia of M83^+/−^ treated with PFF and control in Part I of this study. Unpaired two-tailed *t* test with Welch’s correlation was used to compare these parameters for forelimbs, hindlimbs, and all limbs with control mice. Differences in limb ataxia, paw area peak stance, swing, stance, stride frequency, and stride length in “young” M83^+/−^+PFF mice, “aged” M83^+/−^−PFF, and age-matched control C57BL/6J mice in Part II of this study was similarly assessed with an unpaired two-tailed *t* test with Welch’s correlation. All statistical analyses were performed using GraphPad Prism version 10.4.1 (GraphPad, Boston, MA, USA).

## RESULTS

### Part I: Gait Dynamics Over Time for Cohort A (PFF-Treated Mice)

**Figure 3.**
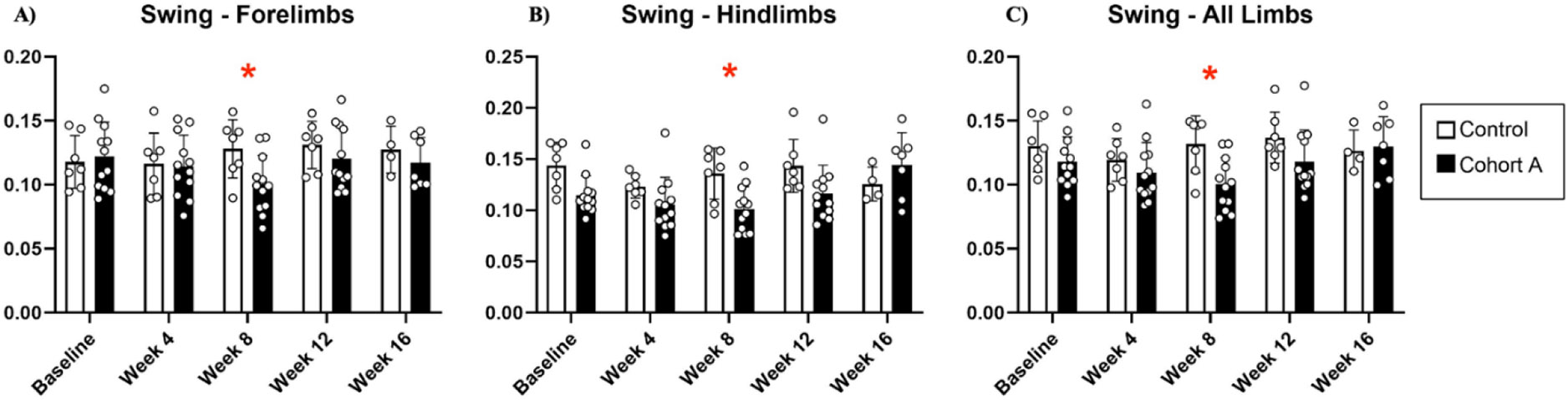
Swing dynamics for cohort A mice from baseline to 16 weeks. Red “*” represents statistically significant differences between the groups.

**Figure 4.**
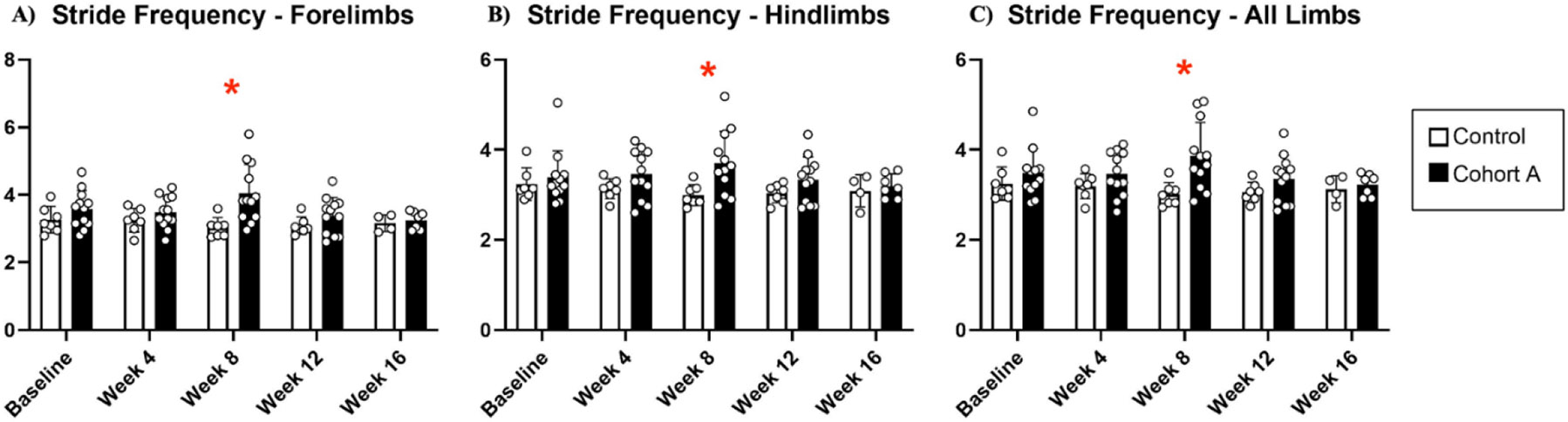
Stride frequency dynamics for cohort A mice from baseline to 16 weeks. Red “*” represents statistically significant differences between the groups.

**Figure 5.**
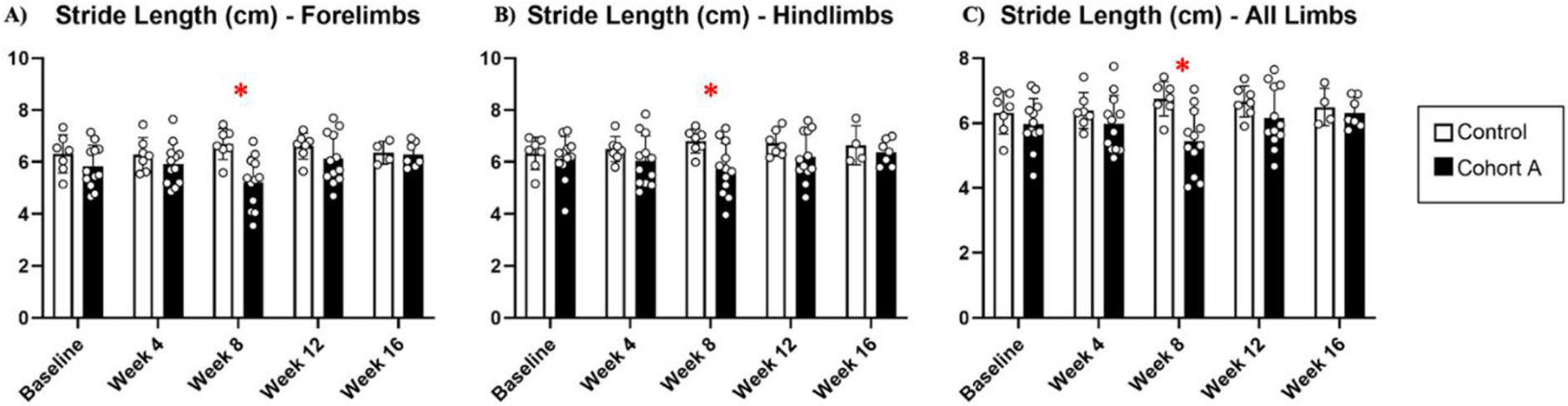
Stride length dynamics for cohort A mice from baseline to 16 weeks. Red “*” represents statistically significant differences between the groups.

**Figure 6.**
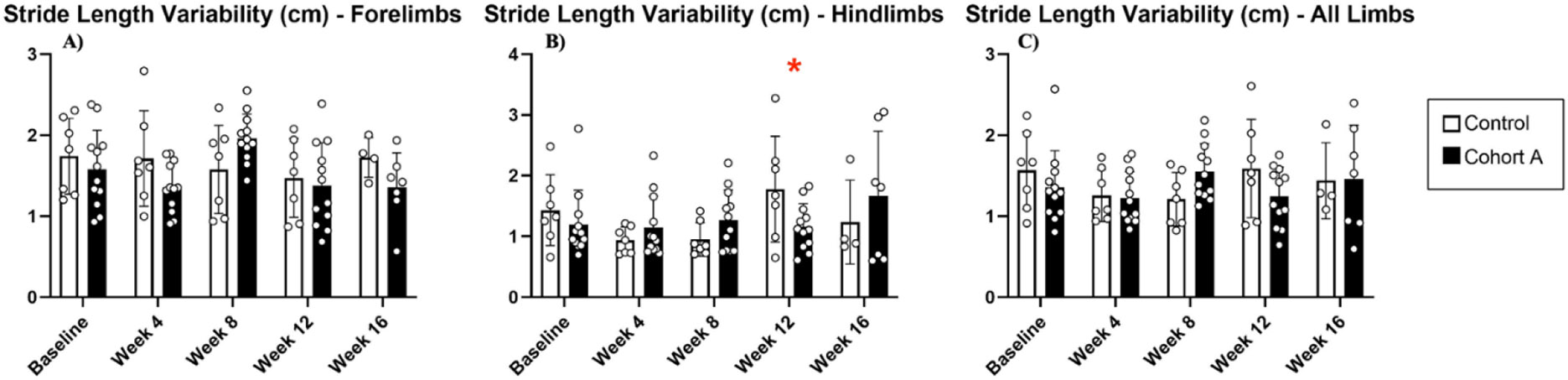
Stride length variability for cohort A mice from baseline to 16 weeks. Red “*” represents statistically significant differences between the groups.

**Figure 7.**
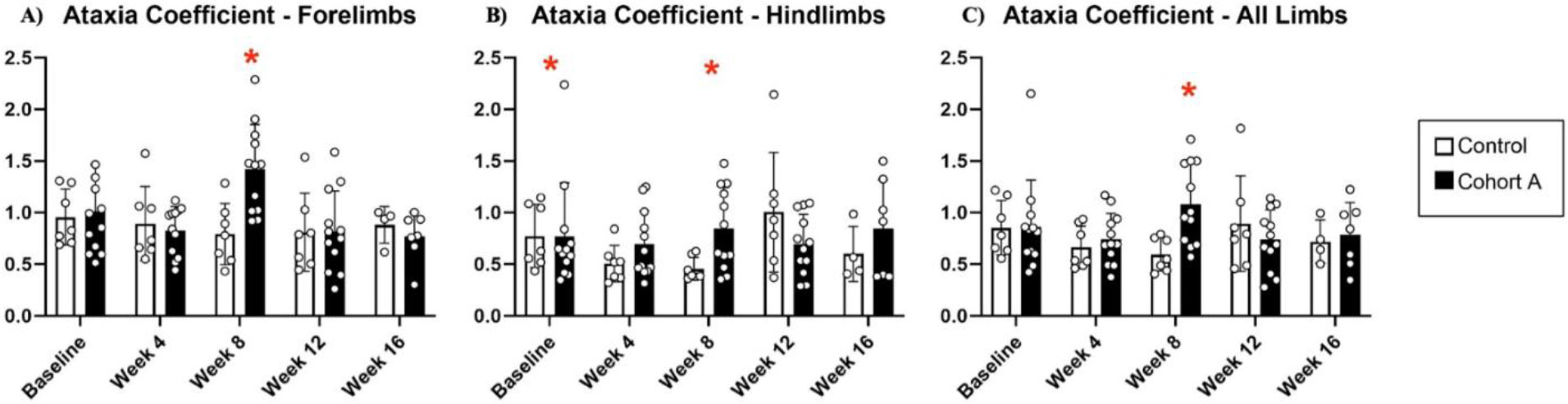
Changes in ataxia coefficient for cohort A mice from baseline to 16 weeks. Red “*” represents statistically significant differences between the groups.

Gait dynamics in this cohort of PFF-injected mice were similar to gait dynamics in the control, saline-treated mice at baseline (Table 1). At week 8, mean (SD) forelimb (Cohort A vs. Control; 0.10 (0.02) vs. 0.13 (0.03), *p* = 0.020), hindlimb (0.10 (0.03) vs. 0.14 (0.04), *p* = 0.006), and all limb (0.11 (0.02) vs. 0.13 (0.03), *p* = 0.007) swing was significantly decreased in PFF-injected mice (Figure 3). Mean (SD) stride frequency for forelimbs (4.0 (0.4) vs. 3.0 (0.5), *p* = 0.009) hindlimbs (3.8 (0.5) vs. 3.0 (0.5), *p* = 0.021), and all limbs (3.9 (0.4) vs. 3.0 (0.5), *p* = 0.010) was significantly increased in PFF-treated mice at 8 weeks (Figure 4). Stride length (mean (SD)) for forelimbs (6.0 (0.5) vs. 7.0 (0.5), *p* = 0.002), hindlimbs (6.5 (0.4) vs. 8.0 (0.6), *p* = 0.016), and all limbs (6.0 (0.4) vs. 7.0 (0.5), *p* = 0.005) was significantly decreased in PFF-treated mice, though this effect was not seen until 12 weeks (Figure 5). Variability in stride length was significantly decreased in PFF-treated mice in the hindlimbs at 12 weeks (1.2 (0.8) vs. 1.9 (1.0), *p* = 0.044, Figure 6). The mean (SD) ataxia coefficient was significantly increased in the forelimbs (1.5 (0.5) vs. 0.9 (0.4) *p* = 0.003), hindlimbs (0.8 (0.6) vs. 0.4 (0.5) *p* = 0.022), and all limbs (1.1 (0.4) vs. 0.6 (0.4) *p* = 0.006) of PFF-treated mice at 8 weeks as compared with control (Figure 7). The most significant changes could be seen at 8 weeks post-PFF treatment. While swing, stride, and other metrics continued to change at 12 and 16 weeks, no statistical significance was observed, for some the animals started to develop motor symptoms due to PFF and were unable to run on the treadmill and were subsequently removed from the study.

### Part II: Comparison of Gait Dynamics between M83^+/−^ Mice and M83^+/−^ Mice at Youth and at An Aged Timepoint

**Figure 8.**
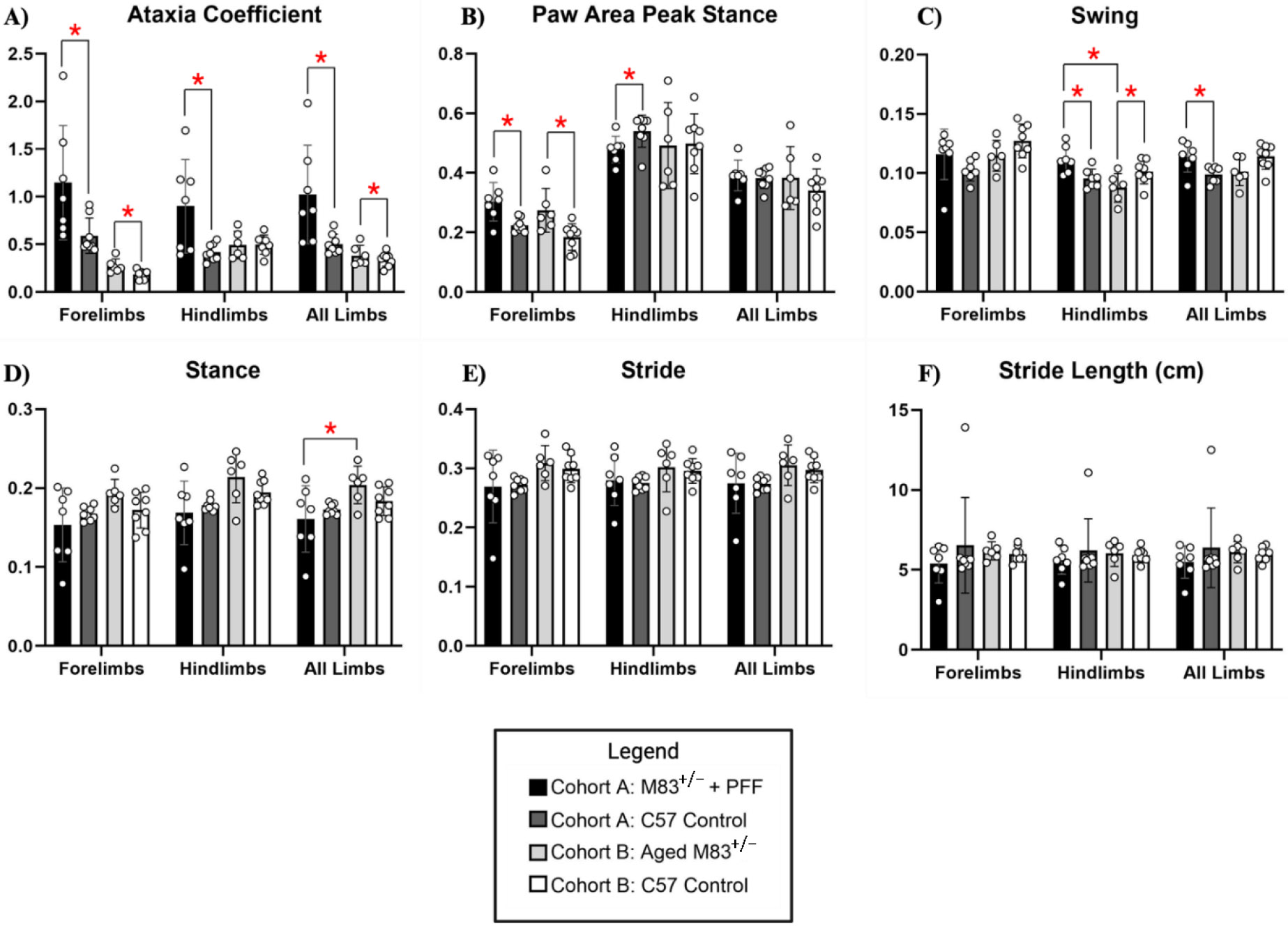
Cohort B Gait Dynamics. (**A**) Ataxia coefficient, (**B**) paw area peak stance, (**C**) swing, (**D**) stance, (**E**) stride, and (**F**) stride length (cm) was measured and compared between young 12-13-week-old M83^+/−^+PFF mice, aged 52-53-week-old M83^+/−^ mice, and age-matched C57 control (12-13- and 52-53-weeks-old, respectively). Red “*” represents statistically significant differences between the groups.

The mean (SD) ataxia coefficient was significantly greater in the forelimbs (1.1 (1.0) vs 0.6 (0.5) *p* = 0.027), hindlimbs (0.9 (1.0) vs. 0.3 (0.2) *p* = 0.016), and all limbs (1.0 (1.0) vs. 0.3 (0.5) *p* = 0.015), of young M83^+/−^ as compared to age-matched C57BL/6J control mice (Figure 8A). Ataxia was similarly significantly increased in the forelimbs (0.3 (0.5) vs 0.2 (0.4) *p* = 0.002) and all limbs (0.5 (1.0) vs. 0.4 (0.2) *p* = 0.008) of aged M83^+/−^ mice as compared to age-matched C57BL/6J control (Figure 8A). Forelimb paw area peak stance (mean (SD)) was significantly increased for young M83^+/−^ (0.35 (0.5) vs. 0.2 (0.4) *p* = 0.006) and aged M83^+/−^ mice (0.3 (0.5) vs. 0.19 (0.5) *p* = 0.015) when compared to their respective age-matched C57BL/6J control mice (Figure 8B). However, hindlimb paw area peak stance was found to be significantly decreased in young M83^+/−^ (0.45 (0.2) vs. 0.55 (0.2) *p* = 0.033) as compared to C57BL/6J control (Figure 8B). Mean (SD) hindlimb swing was found to be significantly increased in young M83^+/−^ mice as compared to both age- matched control (0.11 (0.5) vs. 0.09 (0.2) *p* = 0.036) and aged M83^+/−^ mice (0.11 (0.5) vs. 0.08 (0.3), *p* = 0.015, Figure 8C). Aged M83^+/−^ mice demonstrated significantly lower hindlimb swing as compared to age-matched C57 control (0.08 (0.3) vs. 0.09 (0.2) *p* = 0.044, Figure 8C). For all limbs, swing was significantly increased in young M83^+/−^ as compared with control (0.12 (0.5) vs. 0.10 (0.3), *p* = 0.026, Figure 8C). Mean (SD) stance was significantly increased in aged M83^+/−^ mice as compared with young M83^+/−^ (0.21 (1.0) vs. 0.18 (0.6) *p* = 0.049, Figure 8D). Additional gait dynamic results for this cohort are presented in Table 2.

## DISCUSSION

Murine models that replicate PD are invaluable scientific tools that can help improve our understanding of PD pathogenesis and treatment. Previous studies have been performed to demonstrate widespread α-syn spread in M83^+/−^ mice following PFF injection, but the motor assessments largely evaluate symptoms of ataxia [20]. The current work elaborates on previous foundational research and provides a more comprehensive assessment of motor dysfunction in a relatively understudied murine model of PD.

M83^+/−^ mice treated with PFF demonstrate significant gait abnormalities consistent with features of PD. Significant reductions in stride length accompanied by increased stride frequency mimic shuffling of gait commonly observed in PD [21,22]. Similarly, reduced swing time in PFF-treated mice reflects rigidity and bradykinesia, both of which are defining characteristic of PD associated with reduced movement speed and difficulty initiating and maintaining movements [23-25]. The increased ataxia coefficient in the forelimbs and hindlimbs indicates impaired motor coordination and instability, paralleling the postural instability seen in PD [26,27]. These changes in gait dynamics become more pronounced over time, as shown by the delayed onset of reduced stride length at 12 weeks, which aligns with the progressive nature of PD and may reflect the accumulation of α-synuclein aggregates.

Our findings also highlight regional and age-related differences in motor dysfunction. The lack of significant changes in forelimb swing duration, compared with the more pronounced hindlimb deficits, suggests that motor impairments in this model may vary regionally, a phenomenon often observed in PD [28]. Additionally, aged M83^+/−^ mice exhibit more severe gait abnormalities compared with younger counterparts, consistent with the greater prevalence and severity of PD symptoms in older populations [28]. Alterations in paw area peak stance, with increased forelimb stance and decreased hindlimb stance, further suggest asymmetrical motor dysfunction, a hallmark of early PD [29-31]. Reduced variability in stride length underscores rigidity and diminished adaptability in movement, both prominent features of PD [23] As such, these results validate the PFF-treated M83^+/−^ murine model as a tool for studying PD-related motor symptoms and provide a foundation for developing therapeutic strategies targeting gait and postural control.

Gait disturbances, a hallmark of PD, have been increasingly recognized as potential therapeutic targets through the use of gait variability indices [3,32]. Homozygous M83^+/−^ mice show weight loss, gait abnormalities, balance/coordination deficits, and spatial memory deficits, making them ideal candidates for gait research [20]. The findings from this study provide quantitative evidence that builds upon previous research, supporting the utility of the M83^+/−^ mouse model for investigating motor impairments in PD. By characterizing progressive and region-specific motor deficits, this work emphasizes the importance of detailed gait analysis in preclinical models to improve understanding of disease mechanisms. These results can inform the development of strategies to address motor symptoms and assess the efficacy of potential therapeutic interventions. While the use of DigiGait^™^ is not a novel technique, applying it to quantify gait metrics in a specific strain commonly used in PD experiments provides a valuable framework for future investigations. This approach can help evaluate the effects of various treatments on motor function in the M83^+/−^ model, enabling a more detailed understanding of therapeutic outcomes in PD.

This study is not without limitations. For one, the small sample size employed in this analysis limits the external validity and generalizability of our results. In general, gait dynamics in this mouse model were difficult to study. PFF injection resulted in aggressive motor phenotype and high mortality, and consequently, many of the animals had difficulty walking on the treadmill during later timepoints after PFF injections. These toxicities have been previously reported in the literature but remain limited to one study for M83^+/−^ mice [33]. Despite these limitations, the present study lays the foundation for future research into gait dynamics and variability in mouse models of PD.

Future research strategies should address the limitations and expand on the findings of this study. First, increasing the sample size and ensuring a balanced representation of sex and age groups would improve the generalizability and robustness of the results. Studies could also explore the use of additional motor assessment tools beyond the DigiGait^™^ system to provide complementary data on motor function, such as open-field locomotion tests or balance beam analyses. To address the challenges posed by the aggressive motor phenotype and high mortality observed after PFF injections, future studies could investigate the use of modified injection protocols or lower doses of PFF to reduce toxicity while preserving the ability to induce α-synuclein pathology. Alternative methods to model PD, such as targeted genetic modifications or other pathological inducers, could also be considered. Finally, postmortem analyses of striatal dopamine should be performed to correlate observed gait differences with brain observed pathology.

## CONCLUSIONS

M83^+/−^ treated with PFF and aged M83^+/−^ mice without PFFs show significant gait disturbances, including shortened stride length, and increased stride frequency, and increased ataxia, all characteristic of patients with PD. The results of this study effectively contribute to the growing body of literature supporting the use of the M83^+/−^ model for clinical PD research.

## Figures and Tables

**Table 1.** Cohort A Gait Dynamics. Values are reported as *p*-values. Mean gait dynamic metrics for Cohort A (N = 7) were compared with control. “All” refers to the average of all four limbs.

	Baseline			4 Weeks			8 Weeks		
Parameter	All	Forelimbs	Hindlimbs	All	Forelimbs	Hindlimbs	All	Forelimbs	Hindlimbs
Swing	0.213	0.722	**0.008**	0.343	0.872	0.121	**0.007**	**0.020**	**0.006**
Stride Frequency	0.350	0.202	0.577	0.191	0.268	0.156	**0.010**	**0.009**	**0.021**
Stride Length (cm)	0.341	0.213	0.608	0.290	0.333	0.276	**0.005**	**0.002**	**0.016**
Stride Length Variability (cm)	0.357	0.494	0.395	0.855	0.099	0.307	0.051	0.061	0.138
Ataxia Coefficient	0.950	0.767	0.976	0.474	0.618	0.178	**0.006**	**0.003**	**0.022**

	12 Weeks			16 Weeks		
Parameter	All	Forelimbs	Hindlimbs	All	Forelimbs	Hindlimbs
Swing	0.107	0.339	0.052	0.814	0.413	0.305
Stride Frequency	0.176	0.206	0.156	0.562	0.595	0.567
Stride Length (cm)	0.211	0.237	0.195	0.596	0.799	0.471
Stride Length Variability (cm)	0.138	0.716	**0.044**	0.960	0.151	0.496
Ataxia Coefficient	0.381	0.971	0.134	0.716	0.429	0.364

Note: The bold indicates those metrics which are statistically significant.

**Table 2.** Cohort B Gait Dynamics. Values are reported as *p*-values. Mean git dynamic metrics for 12-13-week-old M83^+/−^+PFF mice (N = 7) were compared with Aged M83^+/−^ (N = 6) and C57 control mice (N = 8). Aged 53-54-week-old M83^+/−^ mice were similarly compared with C57 control mice (N = 8). “All” refers to the average of all four limbs.

	M83^+/−^+PFF vs. C57 Control			Aged M83^+/−^ vs. C57 Control			M83^+/−^+PFF vs. Aged M83^+/−^		
Parameter	All	Forelimbs	Hindlimbs	All	Forelimbs	Hindlimbs	All	Forelimbs	Hindlimbs
Swing	**0.0259**	0.0787	**0.0356**	0.0540	0.1175	**0.0444**	0.1292	0.9008	**0.0154**
Stance	0.4345	0.3982	0.5200	0.0884	0.722	0.9625	**0.0486**	0.0697	0.1799
Stride	0.9560	0.8931	0.7495	0.6120	0.5179	0.7236	0.2380	0.1801	0.3742
Stride Length (cm)	0.3939	0.3617	0.4499	0.5875	0.5000	0.6948	0.2286	0.1732	0.3591
Paw Area Peak Stance	0.6874	**0.0059**	**0.0334**	0.3952	**0.0147**	0.9228	0.8594	0.4572	0.8233
Ataxia Coefficient	**0.0154**	**0.0266**	**0.0164**	**0.0078**	**0.0024**	0.1411	0.4223	0.4102	0.5124

Note: The bold indicates those metrics which are statistically significant.

## Data Availability

All data generated from the study are available in the manuscript or supplementary files.

## ABBREVIATIONS

PD: Parkinson’s Disease
PFF: α-syn preformed fibrils
